# The effect of rhythmic stimuli with spatial information on sensorimotor synchronization: an EEG and EMG study

**DOI:** 10.3389/fnins.2024.1448051

**Published:** 2024-10-04

**Authors:** Huanqing Zhang, Jun Xie, Qing Tao, Zengle Ge, Yu Xiong, Guanghua Xu, Min Li, Chengcheng Han

**Affiliations:** ^1^School of Mechanical Engineering, Xi'an Jiaotong University, Xi'an, China; ^2^School of Mechanical Engineering, Xinjiang University, Ürümqi, China

**Keywords:** sensorimotor synchronization, rhythmic stimuli, neural oscillations, cortico-muscular coherence, spatial information

## Abstract

**Introduction:**

Sensorimotor synchronization (SMS) is the human ability to align body movement rhythms with external rhythmic stimuli. While the effects of rhythmic stimuli containing only temporal information on SMS have been extensively studied, less is known about how spatial information affects SMS performance. This study investigates the neural mechanisms underlying SMS with rhythmic stimuli that include both temporal and spatial information, providing insights into the influence of these factors across different sensory modalities.

**Methods:**

This study compared the effects temporal information and spatial information on SMS performance across different stimuli conditions. We simultaneously recorded the electroencephalogram (EEG), the electromyogram (EMG), and behavioral data as subjects performed synchronized tapping to rhythmic stimuli. The study analyzed SMS performance under conditions including auditory, visual, and auditory-visual motion stimuli (containing both temporal and spatial information), as well as auditory, visual, and auditory-visual non-motion stimuli (containing only temporal information). Specifically, the research examined behavioral data (i.e., mean asynchrony, absolute asynchrony, and variability), neural oscillations, cortico-muscular coherence (CMC), and brain connectivity.

**Results:**

The results demonstrated that SMS performance was superior with rhythmic stimuli containing both temporal and spatial information compared to stimuli with only temporal information. Moreover, sensory-motor neural entrainment was stronger during SMS with rhythmic stimuli containing spatial information within the same sensory modality. SMS with both types of rhythmic stimuli was found to be dynamically modulated by neural oscillations and cortical-muscular coupling in the beta band (13-30 Hz).

**Discussion:**

These findings provide deeper insights into the combined effects of temporal and spatial information, as well as sensory modality, on SMS performance. The study highlights the dynamic modulation of SMS by neural oscillations and CMC, particularly in the beta band, offering valuable contributions to understanding the neural basis of sensorimotor synchronization.

## 1 Introduction

The living environment is rich with rhythmic stimuli, such as music and dance, to which humans often unconsciously adapt their movement rhythms. This ability to synchronize body movements with stimulus rhythms is known as sensorimotor synchronization (SMS) (Blecher et al., 2016; Repp and Su, 2013). SMS facilitates adaptation to environmental changes and enhances performance in daily activities. Additionally, SMS plays a crucial role in the rehabilitation of motor impairments caused by conditions such as Parkinson's disease, stroke, and traumatic brain injury (Forte et al., 2021; Ghai and Ghai, 2019; Thompson et al., 2021).

SMS is influenced by various stimulus properties, particularly the sensory modalities involved (Repp and Su, 2013). Previous research has examined rhythmic visual stimuli, auditory stimuli, and multisensory stimuli (Repp and Su, 2013; Whitton and Jiang, 2023; Repp, 2005). A well-established finding is that, with discrete stimuli, SMS is generally more accurate and less variable with rhythmic auditory stimuli compared to rhythmic visual stimuli. This is attributed to the superior temporal resolution of the auditory system and the stronger sensory-motor coupling associated with auditory stimuli (Whitton and Jiang, 2023; Lorås et al., 2012). In contrast, the visual system is less tightly coupled to the motor system compared to the auditory system (Conway and Christiansen, 2005).

While the auditory system excels in processing temporal information, the visual system is better suited for spatial information (Chen and Vroomen, 2013; Spence and Squire, 2003; Ernst and Bülthoff, 2004). The spatial-temporal structure of stimuli affects SMS performance (Armstrong and Issartel, 2014; Gu et al., 2020; Hove et al., 2013, 2010). Incorporating spatial information through periodic motion in visual stimuli can enhance SMS compared to traditional flash stimuli that provide only temporal information (Gu et al., 2020; Hove et al., 2010). However, existing studies have primarily focused on the behavioral mechanisms of visual stimuli with both temporal and spatial information (Gu et al., 2020; Hove et al., 2010). The neural mechanisms underlying SMS in response to such stimuli remain unclear.

Previous studies have typically been of continuous auditory pacers that do not match discrete limb movement patterns (Ono et al., 2016; Zelic et al., 2019). Although the spatial resolution of auditory stimuli is generally lower than that of visual stimuli (Chen and Vroomen, 2013; Ono et al., 2016), it remains unclear whether supplementing rhythmic discrete auditory motion stimuli with spatial information can enhance SMS during discrete limb movements. Additionally, in terms of auditory perception mechanisms, the human auditory dorsal spatial processing pathway (i.e., perception of spatial auditory position and motion) extends from the temporal plane to the inferior parietal lobe, premotor cortex and finally to the prefrontal cortex or inferior frontal cortex (Arnott et al., 2004; Bizley and Cohen, 2013). Therefore, it is important to investigate whether enhancing neural connections between the auditory cortex and motor cortex can improve SMS performance during the perception of rhythmic auditory motion stimuli with both temporal and spatial information.

The perception of multisensory stimuli has been extensively studied, highlighting how spatiotemporal coherence between multiple sensory inputs aids the brain in integrating information from different sensory channels (Ernst and Bülthoff, 2004; Wuerger et al., 2012). By examining the performance and neural mechanisms of sensorimotor synchronization (SMS) under various bimodal conditions containing both temporal and spatial information, we can clarify whether integrating spatiotemporal information from different sensory channels leads to more stable and accurate perceptions. This research provides valuable perspectives for further studies on multisensory integration.

Electroencephalography (EEG) studies during SMS have revealed that neural oscillations can shed light on the cognitive and sensory processes involved in sensory-motor entrainment (Crasta et al., 2018; Nozaradan et al., 2015). A key finding is that neural entrainment induced by periodic stimuli can be captured in EEG as steady-state evoked potential (SSEP) (Nozaradan et al., 2015). SSEP observed during SMS reflect not only exogenous responses to rhythmic inputs but also endogenous processes related to predicting the timing of upcoming stimuli (Nozaradan et al., 2016). Furthermore, SSEP can be instrumental in exploring the mechanisms of coupling between motor and sensory activities during SMS (Nozaradan et al., 2015). This study aims to investigate whether rhythmic stimuli containing spatial information enhance sensory-motor neural entrainment during SMS by examining the differences in SSEP responses between rhythmic motion stimuli (containing both temporal and spatial information) and rhythmic non-motion stimuli (containing only temporal information).

Previous studies have demonstrated that beta-band neural oscillations (13–30 Hz) play a critical role in driving sensory-motor entrainment during sensorimotor synchronization (SMS) (Crasta et al., 2018; Comstock et al., 2021; Varlet et al., 2020; Nijhuis et al., 2021). These oscillations are particularly involved in predicting the onset of rhythmic stimuli, with beta-band power increasing before the onset of each stimulus and decreasing afterward during SMS (Comstock et al., 2021; Fujioka et al., 2009, 2015). While the involvement of beta-band modulation in SMS has been well-documented, the specific role of beta-band activity in SMS with rhythmic stimuli that include spatial information remains unclear. To address this, our study employed time-frequency analyses to examine changes in beta-band neural oscillations during SMS under two conditions: rhythmic motion stimuli (containing both temporal and spatial information) and rhythmic non-motion stimuli (containing only temporal information). By comparing different stimulus conditions, we aimed to examine the modulatory role of the beta-band in SMS when spatial information is incorporated into rhythmic stimuli.

The functional connection between the cortex and muscles can be measured by cortico-muscular coherence (CMC). Studies have demonstrated CMC can reflect the dynamic mechanisms of cortical-muscular interactions during SMS (Nijhuis et al., 2021). Previous research on SMS with rhythmic auditory stimuli has highlighted the critical role of beta-band neural oscillations in cortico-muscular coupling (Varlet et al., 2020; Nijhuis et al., 2021). In the present study, we employed CMC to investigate whether the dynamics of cortico-muscular coupling differ during SMS with rhythmic stimuli containing spatial information compared to those containing only temporal information. Moreover, brain network analyses were used to explore the interactions between specific functional areas and how these interactions are modulated by cognitive tasks (Baravalle et al., 2019). In the present study, this study analyzed the information flow between brain regions during SMS under various conditions, aiming to uncover the interaction and coupling mechanisms involved in SMS with rhythmic stimuli that incorporate spatial information.

The aim of this study was to explore the neurophysiological mechanisms underlying sensorimotor synchronization (SMS) with rhythmic stimuli that contain spatial information. In the present study, we designed periodic visual and/or auditory motion and non-motion stimuli to assess their impact on SMS. Synchronous finger-tapping aligned with rhythmic stimuli is the main paradigm for studying SMS (Repp and Su, 2013). This study used behavioral analysis, SSEP, time-frequency analysis, CMC, and brain network to assess the brain processing mechanism during SMS (i.e., right index finger-tapping with rhythmic stimuli) with different stimuli conditions. We hypothesized that rhythmic stimuli containing spatial information would more effectively facilitate SMS compared to stimuli lacking spatial information. This facilitation is expected to be evident through increased SSEP amplitudes and enhanced sensory-motor entrainment in motor areas. Specifically, we predicted that adding spatial cues to rhythmic stimuli would lead to more precise timing and coordination of motor responses, reflecting a more robust neural representation of the stimuli, which in turn would improve the accuracy of motor outputs. Furthermore, we anticipated that neural oscillations and cortico-muscular coupling during SMS would be dynamically modulated in the beta band (13–30 Hz). Beta-band activity is closely linked to the anticipation and timing of rhythmic events. We expected to observe an increase in beta-band power just before the onset of each rhythmic stimulus, indicating the brain's preparation for movement. This anticipatory increase would likely be followed by a decrease in beta-band power once the movement is initiated, corresponding to the execution phase of SMS. Such modulation patterns in the beta-band could indicate dynamic coupling between sensory and motor systems, emphasizing the role of beta oscillations in coordinating the temporal aspects of motor actions. Together these analyses, this study aims to uncover the neural mechanisms behind the integration of temporal and spatial information in SMS. These findings provide deeper insights into the neurophysiological basis of this fundamental human ability.

## 2 Materials and methods

### 2.1 Subjects

Fifteen right-handed paid subjects (age range: 20–26 years old) with normal or corrected-to-normal vision, and normal hearing (all subjects were assessed by pure-tone audiometry and had normal hearing, and had no music or dance training), participated in the experiments. The experiments were undertaken in accordance with the recommendations of the Declaration of Helsinki. After fully explaining the experimental procedure to all subjects, each subject signed a written informed consent form in accordance with the guidelines approved by the Institutional Review Committee of Xi'an Jiaotong University.

### 2.2 Stimulation paradigm

During the experiment, three visual and/or auditory stimuli containing both spatial and temporal information [i.e., rhythmic auditory motion stimuli (RAMS), rhythmic auditory-visual motion stimuli (RAVMS), and rhythmic visual motion stimuli (RVMS)] and three visual and/or auditory stimuli containing only temporal information [i.e., rhythmic auditory stimuli (RAS), rhythmic auditory-visual stimuli (RAVS), and rhythmic visual stimuli (RVS)] were presented to the subjects. In this study, spatial information was introduced by varying the spatial location of the stimuli at a specific frequency to create the perception of movement (i.e., apparent motion).

**Figure 1:**
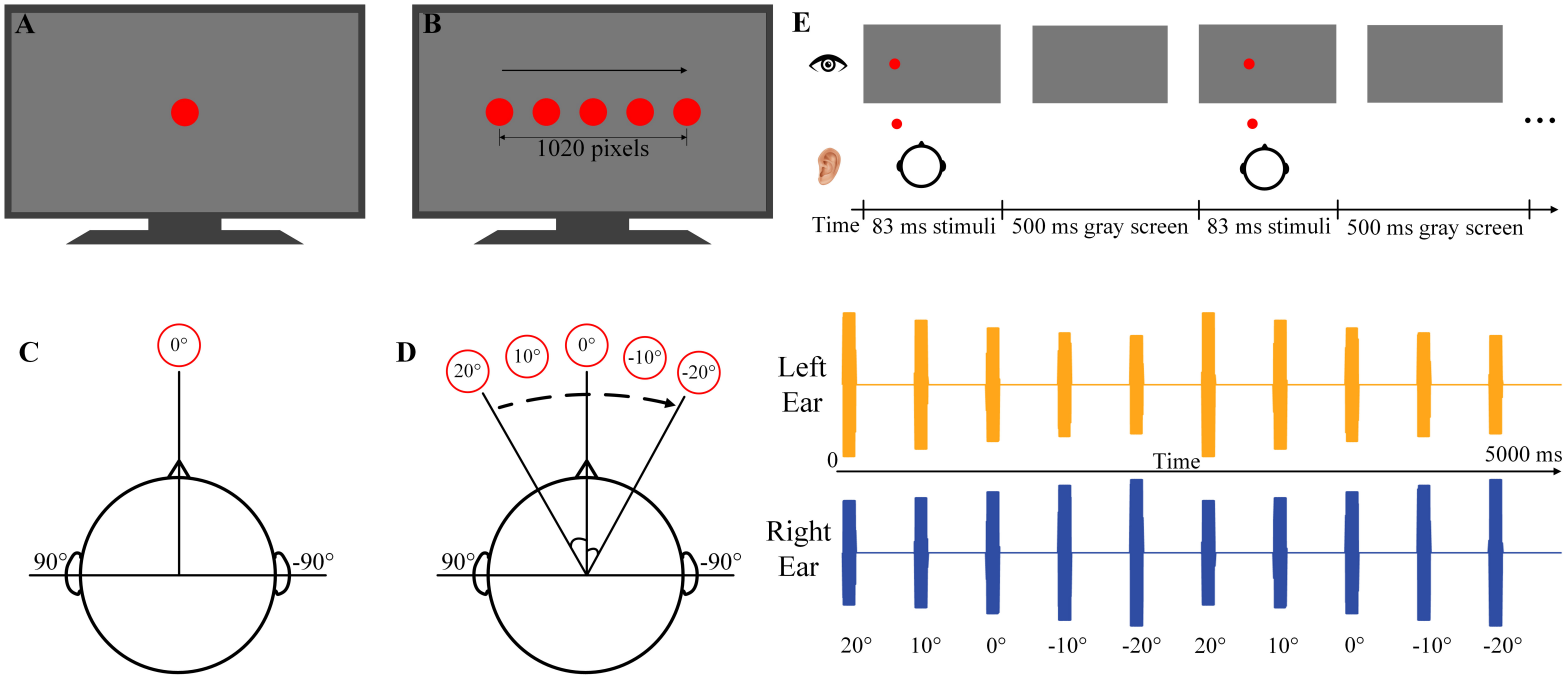
Schematic diagrams of the stimulation paradigms. **(A)** The RVS paradigm. **(B)** The RVMS paradigm. **(C)** The RAS paradigm. **(D)** The RAMS paradigm. The left side of **(D)** is the RAMS schematic diagram, and the right side of **(D)** is the left and right channel audio data waveforms of the spatial location. **(E)** The RAVMS paradigm.

All six stimuli were created in the MATLAB (www.mathworks.com) using Psychophysical Toolbox (Brainard and Vision, 1997). Studies have shown that 2 Hz is the preferred tempo not only for rhythm generation but also for rhythm perception (Bauer et al., 2015; Varlet et al., 2020). Therefore, 2 Hz was selected as the rhythmic stimulus frequency in this study. The visual stimuli consisted of red dots with a diameter of 150 pixels, presented for 83 ms (5 frames) every 500 ms (i.e., 2 Hz). The visual stimuli were displayed on a 27-inch LCD screen with a refresh rate of 60 Hz and a resolution of 1920 × 1,080 pixels (ASUS, China). The background of the LCD screen is black. For RVS, the visual stimuli were always presented in the center of the LCD screen (Figure 1A). For the RVMS, the visual stimuli were presented unidirectionally at five spatial locations in sequence from left to right (Figure 1B).

The auditory stimuli consisted of a 500 Hz pure tone with a sampling rate of 44.1 kHz, presented 83 ms (including a 5 ms linear fade-in and fade-out) for every 500 ms (i.e., 2 Hz). Auditory stimuli were presented by an integrated audio decoder/amplifier of Monitor 09 (MUSILAND, China) and a SENNHEISER IE80S in-ear headphones (SENNHEISER, Germany). The average level of auditory stimuli was set to approximately 60 dB sound pressure level (SPL) measured by acoustic-multi-channel noise testing system (SoundFree, China). For RAMS, spatial virtual audio was generated by means of a head-related transfer function (HRTF). Five spatial locations were selected, where the horizontal angles were 20°, 10°, 0°, −10° and −20° respectively in the horizontal plane and 0° in the elevation angle (Figure 1D). The pure tone was filtered through the HRTF data of the five spatial positions to obtain five sets of spatial audio data. The spatial audio was presented at these five positions in order from left to right (Figure 1D). Moreover, the right side of the Figure 1D shows the audio waveforms at each position in the RVMS. For the RAS, the auditory stimuli were always presented at an elevation angle of 0° and a horizontal angle of 0° (Figure 1C). For bimodal stimuli, this study used the Psychophysical Toolbox to synchronize the presentation of auditory stimuli and visual stimuli. RAVS was achieved by simultaneous presentation of RAS and RVS, and RAVMS was achieved by simultaneous presentation of RAMS and RVMS (Figure 1E).

### 2.3 Experimental procedure

Before the experiment began, each subject read and signed the informed consent form and was briefed on the experimental tasks and procedures. The experiments were conducted in a dimly lit, soundproof room. To explore differences in subjects' SMS abilities on the six stimuli, a finger-tapping task was used. Throughout the experiment, subjects were asked to sit in a chair 50 cm in front of the monitor. Subjects were instructed to tap the space bar on the keyboard with their right index finger in synchrony with the rhythmic stimuli (Whitton and Jiang, 2023). Subjects wore headphones during all trials to prevent outside sounds from interfering with the task. Each subject completed six blocks including RAMS, RAS, RAVMS, RAVS, RVMS, and RVS. Each block consisted of four trials, with each trial lasting 18 s. Subjects rested for 1 min at the end of each trial. The six stimuli were presented in random order during the experiment. Additionally, subjects were given a 5-min break every 15 min. Thus, the total duration of the experiment was ~70 min, including equipment preparation.

### 2.4 EEG and EMG recording

The experiments utilized the g.USBamp system (g.tec, Graz, Austria) to simultaneously record EEG and electromyogram (EMG) at a sampling rate of 1,200 Hz. EEG was recorded with 28 electrodes (i.e., F3, Fz, F4, C6, FT7, FC3, FCz, FC4, FT8, T7, C5, C3, C1, Cz, C4, T8, TP7, CP3, CPz, C2, CP4, TP8, P4, P3, PO3, PO4, O1, and O2) placed according to the International 10–20 system. All scalp channels were referenced to the left earlobe (A1), and the ground electrode was in the frontal position (Fpz). After cleansing the skin with 75% alcohol to reduce surface impedance, EMG was recorded from the right flexor digitorum superficialis (FDS) using a pair of electrodes arranged in a belly-tendon montage on the subjects' right forearm (Kong et al., 2010).

### 2.5 Behavior analyses

Asynchrony, absolute asynchrony, and variability are fundamental indicators in SMS studies that use tapping as a response measure (Repp and Su, 2013). Asynchrony is defined as the difference between the time of the tap and the start time of the corresponding event in the stimulus rhythm. A positive asynchrony indicates that the tap lags the stimulus onset, while a negative asynchrony indicates that the tap occurs before the stimulus onset. Absolute asynchrony refers to the absolute value of the time difference between each tap and the rhythmic stimulus, representing the accuracy of SMS without being influenced by different response strategies. Variability is the standard deviation (SD) of asynchrony and is used to estimate the stability of subjects' synchronous coordination. Absolute asynchrony >50% of the stimulus cycle (i.e., 250 ms) was identified as an outlier and the SMS cycle was removed from the analysis. And the first three cycles at each trial were removed. To ensure that each stimuli condition has the same number of SMS cycles, each stimuli condition (i.e., RAMS, RAS, RAVMS, RAVS, RVMS, and RVS) has 120 cycles of data.

### 2.6 EEG and EMG analyses

#### 2.6.1 Preprocessing

Since this study included four trials per stimulus condition, with each stimulus lasting 18 s at a frequency of 2 Hz, a total of 144 cycles were recorded per stimulus. The first three cycles of each trial, along with cycles containing abnormal behavioral data, were excluded. Additionally, to ensure consistency in the number of SMS cycles across all stimuli conditions, any excess cycles were removed from the end of each trial's data, resulting in exactly 120 cycles for subjects whose data initially exceeded this number after the exclusions.

The first three cycles of data (i.e. 1.5 s) from each trial were first removed to avoid the effect of transient evoked potentials associated with the onset of stimulation and the onset of the tapping movement (Nozaradan et al., 2015). EEG and EMG data were processed using MATLAB (www.mathworks.com). A 4th order 48–52 Hz notch Butterworth filter was applied to remove power-line interference. EEG data were further band-pass filtered using a 4th order Butterworth filter with cut-off frequencies of 1 Hz and 40 Hz. Independent component analysis (ICA) in EEGLAB (Delorme and Makeig, 2004) was used to remove eye movement artifacts. The EMG data were band-pass filtered using a 4th order Butterworth filter with cut-off frequencies of 1 Hz and 190 Hz to retain low-frequency information for steady-state response analysis. For CMC analysis, the EMG data were band-pass filtered using a 4th order Butterworth filter with cut-off frequencies of 10 Hz and 195 Hz to remove motion artifacts and noise (Nijhuis et al., 2021). EEG and EMG data were down sampled to 500 Hz.

#### 2.6.2 Frequency response

SSEP was analyzed using 5-s segments of EEG data for each stimulus condition. The 5-s duration was chosen to ensure effective evocation of the SSEP and to maintain appropriate frequency resolution. For each subject and stimulus, the EEG and EMG data were segmented into 5-s windows with no overlap, and then the data were superimposed and averaged to improve the signal-to-noise ratio (SNR) of the SSEP and the frequency response of EMG. The averaged waveforms were subsequently transformed into the frequency domain using the Fast Fourier Transform (FFT), resulting in a spectrum with a frequency resolution of 0.2 Hz. Moreover, the SSEP of EEG is lower than that the frequency response of EMG, and the SSEP is at 2 Hz, which makes it susceptible to interference from low-frequency components. To avoid interference from low-frequency noise, this study used the SNR to represent the intensity of the SSEP of EEG. SNR was defined as the ratio of the FFT amplitude at a given frequency *f* to the average amplitude of the SSEP in the adjacent 2 Hz band [*f*-1, *f* +1]. The SNR was calculated by the following equation:


$$\begin{array}{l} SNR\left(f\right)=a\left(f\right)/\{\frac{1}{10}\overset{l=5}{\underset{l=-5,l\neq 0}{\sum }}a\left(f+l\cdot \text{Δ}f\right) \end{array}$$

Where a(*f* ) represents the power spectrum amplitude at the frequency *f* . Δ*f* is the frequency resolution of 0.2 Hz.

#### 2.6.3 Time-frequency analyses

Time-frequency analysis can reflect time-domain and frequency-domain characteristics. In this study, time-frequency analysis was performed on 1-cycle data. The short time Fourier transform (STFT) based on the FFT was used to perform time-frequency analysis of EEG and EMG power on each subject. The window length was fixed at 0.25 s. This window size was chosen to avoid overlap between successive stimuli and to make it possible to examine within-cycle modulation with sufficient temporal and frequency resolution (Nijhuis et al., 2021). Time-frequency analyses were performed for each trial, then the time-frequency data were superimposed to obtain the time-frequency data based on induced power.

CMC is a frequency-domain analysis method to investigate the coupling between the simultaneously recorded cortical EEG signals and the EMG signal, during excitatory muscle contraction. In this study, CMC was employed to explore the temporal localized oscillations in EEG and EMG, as well as the dynamic coupling between cortical and muscular activity during SMS. The wavelet coherence can provide higher resolution in the time-frequency domain, thus capturing more subtle dynamic changes. Compared to other traditional CMC coherence measurements, wavelet coherence has an advantage when dealing with non-stationary signals because it can analyse both frequency and time information of the signal (Chavez and Cazelles, 2019). Therefore, the “wcoherence” function in MATLAB was used to perform the CMC analysis to obtain wavelet coherence for each trial. CMC data from each trial were then superimposed to obtain CMC based on induced EEG and EMG data. Dynamic analysis of beta-band (13–30 Hz) power and CMC was conducted based on the time-frequency analysis. For dynamic responses, beta-band power and CMC were analyzed during a stimulus cycle to explore differences in dynamic beta-band power and CMC responses at different stimulation conditions. The present study focused on comparing the differences in motor areas during SMS in different stimulation conditions. Moreover, since all subjects responded by tapping with their right hand, the C3 electrode was selected for analysis because it is located over the left motor cortex, which is responsible for controlling the right hand (Crasta et al., 2018).

#### 2.6.4 Directed transfer function (DTF)

The Directed Transfer Function (DTF) is a brain network analysis method that focuses on causal interactions between brain regions (Kaminski and Blinowska, 1991). The beta band is closely related to motor control in the brain. Analyzing brain connectivity within the beta band can reveal the flow of information between different brain areas, thus helping to further understand the interactions between different areas of the brain during SMS. In the present study, we analyzed information flow between brain areas in the beta-band neural oscillations during SMS at different types using DTF and investigated the interaction and coupling mechanisms between sensory cortex and motor cortex during SMS with rhythmic stimuli containing spatial information.

The eConnectome toolbox (He et al., 2011) was employed to calculate the DTF between EEG channels. The DTF between two EEG channels contains input flow and output flow. The value of the DTF ranges from 0 to 1, and the larger the value the greater the input/output of information flow between the regions of interest. This study calculated the DTF within the beta band for one stimulus cycle of EEG data from all subjects across different stimuli conditions. Additionally, the mean values of input flow and output flow in the left motor area of the brain (i.e., C5, C3, C1, FC3, and CP3) (Nijhuis et al., 2021) were also quantified to compare the differences in connectivity in the motor areas of the brain during SMS with different stimuli.

#### 2.6.5 Statistical analyses

In the present study, ANOVA was used to assess the differences in different stimuli conditions. All pairwise comparisons were adjusted using the Bonferroni correction. Statistical significance was set as *p* < 0.05. The results are expressed as means ± SD.

## 3 Results

### 3.1 Movement synchronization

**Figure 2:**
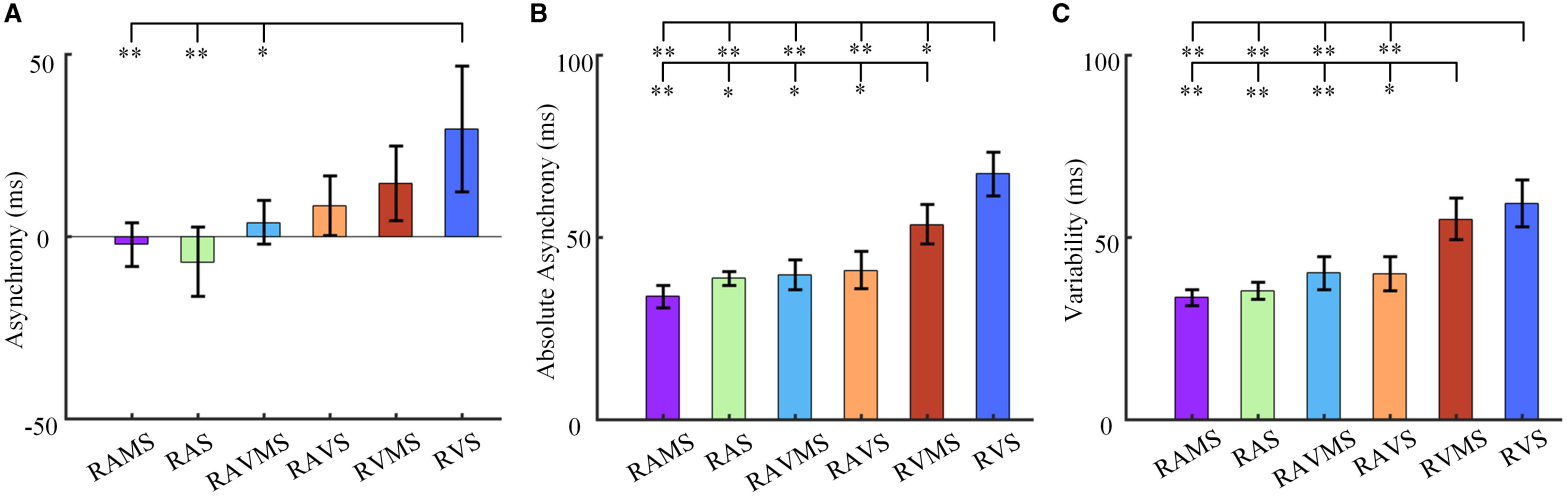
SMS performance. Statistical results of asynchrony **(A)**, absolute asynchrony **(B)**, and variability **(C)** at six stimulation conditions (i.e., RAMS, RAS, RAVMS, RAVS, RVMS, and RVS). The error bar represents the standard error. The ‘*' denotes the multiple comparison results, *p < 0.05 and **p < 0.001.

As shown in Figure 2A, the mean asynchrony of auditory stimuli [i.e., RAMS (−0.002 ± 0.015) and RAS (−0.007 ± 0.023)] were negative values (i.e., tapping earlier than stimulus presentation), whereas the mean asynchrony of auditory-visual stimuli [i.e., RAVMS (0.004 ± 0.015) and RAVS (0.009 ± 0.020)] and visual stimuli [i.e., RVMS (0.015 ± 0.025) and RVS (0.029 ± 0.042)] were positive values (i.e., tapping later than stimulus presentation). A one-way ANOVA was used to analyze differences in the mean asynchrony across different stimuli conditions. Stimuli conditions significantly affected the mean asynchrony of SMS [*F*_(5, 84)_ = 4.31, *p* = 0.001, η^2^ = 0.183]. The multiple comparisons with Bonferroni correction were then performed to show that the mean asynchrony at RVS was significantly different from that at RAMS (*p* = 0.009), RAS (*p* = 0.001) and RAVMS (*p* = 0.046).

In addition, stimuli conditions also significantly affected the absolute asynchrony of SMS [*F*_(5, 84)_ = 20.49, *p* < 0.001, η^2^ = 0.516]. As shown in Figure 2B, the absolute asynchrony at RVS was significantly different from that at RAMS (*p* < 0.001), RAS (*p* < 0.001), RAVMS (*p* < 0.001), RAVS (*p* < 0.001), and RVMS (*p* = 0.007). And the absolute asynchrony at RVMS was significantly different from that at RAMS (*p* < 0.001), RAS (*p* = 0.004), RAVMS (*p* = 0.009), and RAVS (*p* = 0.029).

Figure 2C compares the variability of SMS. Stimuli conditions significantly affects the variability of SMS [*F*_(5, 84)_ = 14.59, *p* < 0.001, η^2^ = 0.432]. The variability at RVS was significantly different from that at RAMS (*p* < 0.001), RAS (*p* < 0.001), RAVMS (*p* < 0.001), RAVS (*p* < 0.001). The variability at RVMS was significantly different from that at RAMS (*p* < 0.001), RAS (*p* < 0.001), RAVMS (*p* = 0.005), RAVS (*p* = 0.004). No significant difference was found in other cases.

These results indicated that auditory and auditory-visual stimuli were superior to visual stimuli in SMS. The SMS performance of RAMS with spatial information was better than RAS, but there was no statistical significance. Moreover, the SMS performance of RVMS with spatial information was superior to RVS.

### 3.2 EEG and EMG responses

**Figure 3:**
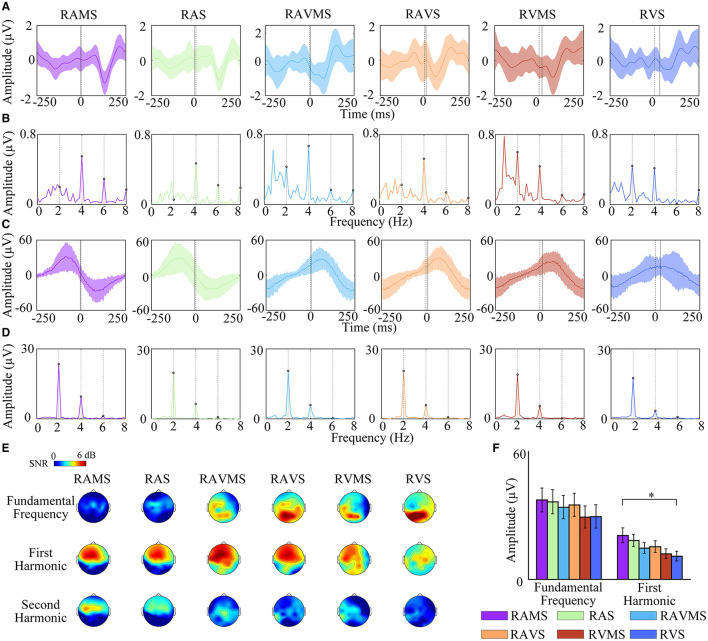
Frequency response analysis of EEG and EMG elicited by RAMS, RAS, RAVMS, RAVS, RVMS, and RVS. A and B: Time-domain waveforms **(A)** and frequency-domain waveforms **(B)** of EEG in the C3 channel. The dashed lines represent stimuli onset time 0, and the solid lines represent the tapping time. **(C, D)** Time-domain waveforms **(C)** and frequency-domain waveforms **(D)** of EMG in the FDS. Shaded areas represented standard deviation. **(E)** Topographic maps of the fundamental, first harmonic, and second harmonic amplitudes of SSEP of EEG. **(F)** Statistical results of frequency component amplitude of frequency response of EMG, including the fundamental frequency and the first harmonic. The error bar represents the standard error.

To investigate the effect of different stimulation conditions on SSEP during SMS, the time-domain and frequency-domain of EEG and EMG elicited by six stimuli during SMS were analyzed in this study (Figure 3). Figures 3A, C depict the time-domain waveforms of the EEG (Figure 3A) in the C3 channel and the EMG (Figure 3C) in the right FDS muscle over the time window of −250 ms to 250 ms averaged over all subjects. The shaded area represented the standard deviation. The time 0 represented the onset of the stimulus. The EEG broadband frequency range is 1 Hz−40 Hz for Figure 3A timeseries. The EMG broadband frequency range is 1 Hz−190 Hz for Figure 3B timeseries. Figures 3B, D show the frequency-domain waveforms of EEG (Figure 3B) and EMG (Figure 3D) in the 0–15 Hz frequency window corresponding to time domain waveforms of Figures 3A, C. To further analyze the distribution of SSEP in scalp at different stimulation conditions, topographic analyses were performed for the SNR of fundamental, first, and second harmonics of SSEP (Figure 3E). SSEP elicited by RAMS and RAS was mainly located in the frontal lobe and the energy of SSEP was concentrated in the first and second harmonics. SSEP elicited by RVMS and RVS was mainly located in the occipital lobe and the energy of SSEP was mainly concentrated in the fundamental frequency. In addition, SSEP elicited by RVMS were also found in the second harmonic at the left frontal-central region. The fundamental frequency of SSEP elicited by RAVMS and RAVS was mainly distributed in the occipital region, while the first and second harmonics of SSEP were mainly distributed in the frontal-central region.

Then this study used a three-factor (stimulation conditions^*^harmonics^*^electrodes) repeated measures-ANOVA to statistically assess the SSEP differences in the motor area during SMS with different stimuli (i.e., RAMS, RAS, RAVMS, RAVS, RVMS, and RVS). Referring to the literature (Nijhuis et al., 2021), electrodes were selected for the left motor area with five electrodes including C1, C3, C5, CP3, and FC3. Harmonics were selected for the fundamental frequency, first, and second harmonics. The results showed that there was a significant effect of stimulation conditions [*F*_(5, 1344)_ = 7.27, *p* < 0.001, η^2^ = 0.0253], harmonics [*F*_(2, 1347)_ = 78.38, *p* < 0.001, η^2^ = 0.1092] on SSEP in the left motor cortex. In contrast, electrodes [*F*_(4, 1345)_ = 2.68, *p* = 0.0503, η^2^ = 0.008] had no significant effect on SSEP in the left motor cortex. There was a significant interaction effect between harmonics and stimulation conditions [*F*_(10, 1339)_ = 9.19, *p* < 0.001, η^2^ = 0.064]. And there were no significant interaction effects between the other factors. For the factor of stimulation conditions, the SNR RAMS (*p* = 0.012), RAS (*p* = 0.0431), RAVMS (*p* < 0.001), RAVS (*p* < 0.001), and RVMS (*p* < 0.001) were significantly higher than RVS in the left motor cortex. For the harmonic factor, SNR was significantly higher for the first harmonic than for the fundamental frequency (*p* < 0.001) and second harmonic (*p* < 0.001). Figure 3D shows that the EMG energy was mainly concentrated in the fundamental and first harmonics, so the harmonics of EMG were selected for analysis in the fundamental and first harmonics. As shown in Figure 3F, one-way repeated measures-ANOVA showed the first harmonic of EMG at RAMS were significantly higher than RVS [*F*_(1, 28)_ = 5.23, *p* = 0.0306]. In other cases, there was no significant difference.

To assess the relationship between the beat-related the frequency response of EMG as well as SSEP and the performance of SMS, we performed correlations between the amplitude of the beat-related SSEP as well as the frequency response of the EMG and the performance of SMS under different stimuli conditions. The mean amplitude of beat-related EMG frequency responses in different stimuli conditions was significantly correlated with the mean asynchrony [*r*_(13)_ = −0.811, *p* = 0.046], variability [*r*_(13)_ = −0.95, *p* = 0.004], and non-significantly correlated with absolute asynchrony [*r*_(13)_ = −0.647, *p* = 0.165]. The beat-related mean SSEP amplitudes in different stimuli conditions were not significantly correlated with mean asynchrony [*r*_(13)_ = −0.214, *p* = 0.684], variability [*r*_(13)_ = −0.356, *p* = 0.489], and absolute asynchrony [*r*_(13)_ = −0.438, *p* = 0.385].

### 3.3 Time-frequency response

**Figure 4:**
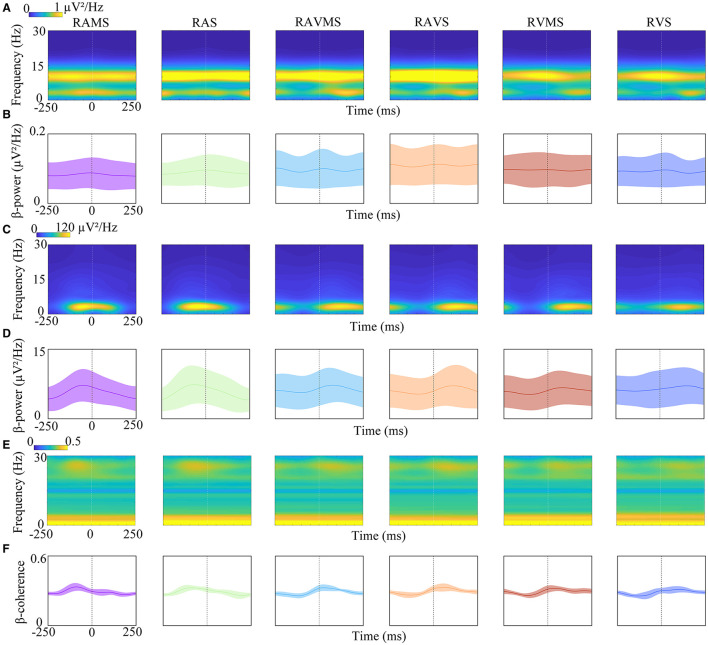
Time-frequency analyses of EEG power **(A, B)**, EMG power **(C, D)**, and EEG-EMG coherence **(E, F)** elicited by RAMS, RAS, RAVMS, RAVS, RVMS, and RVS. **(B, D, F)** EEG beta power **(B)**, EMG beta power **(D)**, and EEG-EMG beta coherence **(F)** dynamic change process with time. Shaded areas represented standard deviation.

To investigate the time-frequency characteristics of response of brain motor area and FDS during SMS at different stimuli, EEG and EMG of one stimulation cycle were analyzed by STFT. Figures 4A, C show the power-based time-frequency plots of the EEG (i.e., C3) and EMG. The time 0 represented the onset of the stimulus. Figure 4A shows that the energy of EEG induced by RAMS, RAS, RAVMS, RAVS, RVS, and RVMS during SMS in the EEG was primarily concentrated between 2 and 15 Hz, with particularly strong energy observed in the alpha-band throughout the cycle. Figure 4C shows that EMG energy induced by RAMS, RAS, RAVMS, RAVS, RVS, and RVMS was mainly concentrated at 2–4 Hz. In terms of time, the EMG energy induced by RAMS and RAS was mainly concentrated at −100–100 ms. The EMG energy induced by RVS and RVMS was mainly concentrated between 0 and 200 ms. The EMG energy induced by RVS and RVMS was mainly concentrated between 50 and 200 ms. In addition, the time distribution of the energy reflects the relationship between the time of tapping and the time of stimulus appearance. And the EMG energy induced by RAMS, RAS, RAVMS, and RAVS was stronger than RVS and RVMS, which was related to the more synchronized and stable taping motion at these four stimuli types (i.e., RAMS, RAS, RAVMS, and RAVS).

To access the differences in beta-band power dynamic change during SMS with different stimulation conditions, the dynamic change of beta-band power of EEG (Figure 4B) and EMG (Figure 4D) were analyzed with one stimulus cycle. The time 0 represented the onset of the stimulus. As shown in Figure 4B, the beta-band power of EEG rises before the stimulus appears and the energy falls after the stimulus appears, but the magnitude of this change is small. A 6^*^50 two-factor ANOVA was performed on the stimulation conditions and time factors (10 ms increments) for the beta-band EEG. The results showed significant effect of the stimuli [*F*_(5, 4494)_ = 21.8, *p* < 0.001, η^2^ = 0.0293] on beta-band power. No significant effect of the time factor [*F*_(49, 4450)_ = 0.14, *p* > 0.05, η^2^ = 0.002] was found on beta-band power. In addition, there was no evidence of significant interaction between stimulus conditions and time factors [*F*_(245, 4254)_ = 0.03, *p* > 0.05, η^2^ = 0.002]. Moreover, A 6^*^50 two-factor ANOVA was performed on the stimulation conditions and time factors (10 ms increments) for the beta-band EMG. The results showed significant effect of stimuli [*F*_(5, 4494)_ = 2.67, *p* = 0.020, η^2^ = 0.135] and time factor on the beta-band power [*F*_(49, 4450)_ = 1.43, *p* = 0.028, η^2^ = 0.232]. But there was no significant interaction between stimulus and time factors [*F*_(245, 4254)_ = 0.62, *p* = 0.999, η^2^ = 0.2698].

To investigate the dynamic coupling mechanism between the brain motor cortex and muscles during SMS with different stimulation conditions, the present study used CMC to analyze the time-frequency coherence between EEG (i.e., C3) and EMG. Figure 4E depicts the time-frequency EEG-EMG coherence plots at different stimulation conditions. The time 0 represented the onset of the stimulus. The results revealed strong frequency coherence below 5 Hz during SMS, which was related to the consistency of stimulation frequency and tapping frequency during SMS. Then the dynamic coupling between cortical muscles at beta-band (13–30 Hz) was further analyzed. Figure 4F shows the dynamics of cortical-muscle coherence in the beta-band during a stimulation cycle. The peak of CMC at RAS and RAMS appeared before the stimulus, while the peak of CMC at the other stimulation conditions (i.e., RAVMS, RAVS, RVS, and RVMS) appeared after the stimulus. This is related to the fact that tapping at RAS and RAMS is earlier than the stimulus, whereas tapping at the other stimulation conditions (i.e., RAVMS, RAVS, RVS, and RVMS) is slower than the stimulus. A 6^*^50 ANOVA on the stimulation conditions and time (10 ms increments) factors for the beta band showed significant effect of the stimulation conditions [*F*_(5, 4494)_ = 10.15, *p* < 0.001, η^2^ = 0.079] and time factor [*F*_(49, 4450)_ = 23.54, *p* < 0.001, η^2^ = 0.172] on the beta-band coherence. There was significant interaction between stimulation conditions and time factors [*F*_(245, 4254)_ = 7.75, *p* < 0.001, η^2^ = 0.2831]. For the stimuli factors, cortical-muscle coherence was significantly higher during SMS with RAMS (*p* = 0.003), RAS (*p* < 0.001), RAVMS (*p* = 0.044), RAVS (*p* < 0.001), and RVMS (*p* < 0.001) than RVS.

### 3.4 Connectivity results

**Figure 5:**
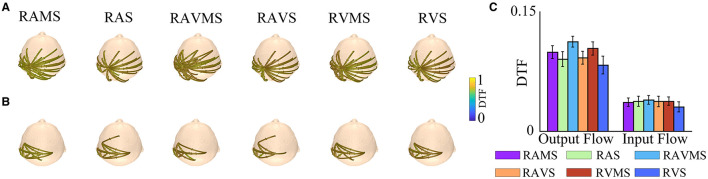
DTF analyses of beta band EEG (−250–250 ms) elicited by RAMS, RAS, RAVMS, RAVS, RVMS, and RVS. **(A)** Left motor area (C1, C3, C5, CP3, and FC3) information output flow. **(B)** Left motor area information input flow. **(C)** Statistical results of DTF of the left motor area, including the input and output flows. The error bar represents the standard error.

To investigate the cortical processing mechanisms during SMS at different stimulation conditions, the present study assessed the intercortical connectivity in the beta band by DTF. Figures 5A, B depict the connectivity between brain regions at SMS with different stimulation conditions, showing only the connections between the left motor cortex channels (C1, C3, C5, CP3, and FC3) and the other channels above 75% of the maximum DTF values in the six stimulation conditions. The arrows on the interpolar connections represent the direction of information flow. Figure 5A shows the information flow output from the left motor cortex and Figure 5B shows the information flow input to the left motor cortex. As can be seen in Figure 5A, the connectivity of the motor cortex is more extensive during SMS with stimuli containing spatial information. Then, to investigate the differences in information transmission in the motor cortex during SMS with different stimuli conditions, we performed a statistical analysis of the input and output flows of the left motor area electrodes (C1, C3, C5, CP3, and FC3) (Figure 5C). A two-factor (stimulation conditions^*^output flow and input flow) ANOVA was performed for statistical analysis. The results showed no significant effect of the stimulation conditions on DTF [*F*_(5, 174)_ = 1.25, *p* = 0.290, η^2^ = 0.020]. There was a significant effect of the two different types of output and input flows on DTF [*F*_(1, 178)_ = 160.45, *p* < 0.001, η^2^ = 0.519]. There was no significant interaction effect between the two factors [*F*_(5, 174)_ = 0.52, *p* = 0.7582, η^2^ = 0.071]. In addition, the output flow in the motor area was significantly higher than the input flow (*p* < 0.001).

## 4 Discussion

The present study examined the effects of spatial information and sensory modality on the SMS (i.e., finger-tapping) performance. The behavioral data (i.e., mean asynchrony, absolute asynchrony, and variability), EEG and EMG responses during SMS with six stimulation conditions (i.e., RAMS, RAS, RAVMS, RAVS, RVMS, and RVS) were investigated. As we predicted, SMS performance was superior for auditory stimuli and auditory-visual stimuli compared to visual stimuli alone. Additionally, visual stimuli containing spatial information (i.e., RVMS) showed superior performance over those containing only temporal information (i.e., RVS). This result aligns with our hypothesis that the inclusion of spatial cues in rhythmic stimuli would facilitate more precise timing and coordination of motor responses. Time-frequency analysis revealed that beta-band power and CMC in the motor cortex followed a consistent dynamic pattern across the different stimuli conditions within each stimulus cycle, though notable dynamic differences were observed. Furthermore, the information output flow in the beta band of motor areas during SMS with spatial information was significantly greater than the information input flow, suggesting a robust neural representation and proactive preparation for movement. These findings corroborate our hypothesis that spatial information in rhythmic stimuli enhances sensory-motor entrainment and highlight the crucial role of beta oscillations in coordinating the temporal aspects of motor actions.

In this study, we designed rhythmic motion stimuli based on the periodic change of spatial position, where the stimulus frequency refers to the rate at which the stimulus changes spatial position discretely. Our study integrates spatial and rhythmic information by stimulating rhythmic changes in spatial position for both auditory and visual stimuli. Unlike traditional visual motion paradigms, which typically use continuous motion, our stimuli are presented at discrete locations. Previous SMS studies achieved motion stimuli by introducing continuous displacement into visual stimuli (Zelic et al., 2016; Comstock et al., 2018). In contrast, unlike continuous auditory and visual pacers (Ono et al., 2016; Zelic et al., 2019), referring to previous study (Whitton et al., 2024), our design discretely presents spatial motion information to further investigate rhythm processing in relation to spatial localization. This method aligns the rhythmicity of visual and auditory stimuli more closely with actual rhythmic motion, enhancing the study of spatial-temporal information effects on SMS. By emphasizing discrete spatial variations, this design allows for a more precise examination of how spatial and rhythmic information interact during perception and action. This approach not only provides a novel perspective for exploring the independence of motion and rhythm perception but also establishes a new experimental framework for understanding their distinct roles in sensory-motor synchronization.

Different strategies are adopted for SMS at different rhythmic sensory stimuli (Repp and Su, 2013; Whitton and Jiang, 2023). SMS with auditory stimuli (i.e., RAMS and RAS) typically exhibits negative mean asynchrony, indicating that participants tend to anticipate and synchronize their responses before the auditory stimuli occur. Conversely, SMS with auditory-visual and visual stimuli (i.e., RAVMS, RAVS, RVMS, and RVS) shows positive mean asynchrony, suggesting that participants adopt a tracking strategy where synchronization occurs as they follow the stimuli. The absolute asynchrony results reveal that SMS accuracy is significantly higher for both auditory and auditory-visual stimuli compared to visual stimuli. Notably, visual stimuli with spatial information (RVMS) also show significantly better accuracy than those with only temporal information (RVS). These findings are consistent with previous behavioral studies (Gu et al., 2020; Zelic et al., 2019). The variability results further highlight sensory channel differences. The variability is significantly higher with visual stimuli compared to auditory and auditory-visual stimuli. This aligns with previous research on sensory processing differences (Whitton et al., 2024; Armstrong and Issartel, 2014), which suggests that auditory stimuli typically offer more pronounced temporal cues, resulting in lower synchronization variability. The spatial aspects of visual stimuli, such as those involving movement or collision, can enhance temporal perception (Hove et al., 2010; Iversen et al., 2015). Our findings support this, demonstrating that visual stimuli with spatial information reduce synchronization variability and errors. The enhanced perception of stimulus onset in such contexts may be due to spatial cues anchoring the timing of movements more effectively (Hove and Keller, 2010). This suggests that incorporating spatial aspects in visual stimuli can improve synchronization performance, similar to how auditory stimuli provide clear temporal cues. Furthermore, auditory stimuli with spatial information (RAMS) were superior to auditory stimuli without spatial information (RAS) in tapping performance, but not significantly different. This may be due to the complexity of perceiving and processing auditory spatial information, which can vary based on individual differences and task demands (Aschersleben, 2002; Blauert, 1997; Kidd et al., 2007). In some cases, the temporal resolution of the auditory system may be sufficient for non-spatial information to provide adequate rhythmic cues, rendering the additional spatial information less impactful. Our results reflect this complexity. Future research could involve more complex motion tasks to further explore the potential advantages of auditory rhythm with spatial information.

Neural entrainment at rhythmic frequencies plays a crucial role in the perception of rhythmic stimuli (Nozaradan et al., 2015; Keller et al., 2014). Rhythmic stimuli trigger periodic neural responses that manifest as SSEP in the EEG (Nozaradan, 2014). This rhythmic entrainment involves oscillations in one brain region synchronizing with oscillations in another, resulting in an interregional oscillatory lock. In this study, EEG was recorded during SMS, not just passive listening, meaning that the SSEP observed were driven by motor tasks rather than mere stimulus perception. Neural oscillations at 2 Hz and its harmonics were detected, indicating synchronization between brain oscillations and rhythmic stimuli. The stimuli conditions notably impacted SSEP in the motor cortex. Our findings indicate that SSEP elicited by auditory, auditory-visual, and RVMS stimuli were significantly stronger in motor cortical areas compared to RVS. Among sensory channels, stronger neural entrainment was observed during SMS with auditory and auditory-visual stimuli. Visual stimuli containing spatial information appeared to enhance entrainment in the sensory-motor cortex and strengthen neural oscillations in the motor cortex. Coupling between sensory and motor-related neural regions modulates neural oscillations, facilitating accurate SMS during rhythmic tasks (Nozaradan et al., 2015). The phase alignment of neural oscillations enhances multisensory response times and supports time prediction and motion planning (Mercier et al., 2015; Gupta and Chen, 2016). Furthermore, the accuracy of SMS is linked to neural mechanisms. The amplitude of SSEP correlates with SMS accuracy, suggesting a potential predictive relationship (Nozaradan et al., 2016; Nave et al., 2022). The poorer SMS performance observed with RVS may be attributed to weaker neural oscillations in the motor cortex. Muscle activity recorded by EMG also showed stronger responses during auditory and auditory-visual stimuli compared to visual stimuli, likely due to the more accurate and stable tapping facilitated by these sensory modalities. SMS performance can also be reflected in the energy of the EMG (Yoles-Frenkel et al., 2016).

In this study, time-frequency analysis was employed to investigate the dynamic differences in neural oscillations during SMS with rhythmic stimuli containing spatial information compared to those containing only temporal information. The motor system's contribution to rhythm perception is reflected in the dynamic modulation of motor cortical activity that occurs in synchrony with external stimuli (Bengtsson et al., 2009). The amplitude modulation of beta-band oscillations in the motor system is commonly associated with the initiation and sustained execution of movements, including the top-down regulation of the sensorimotor system (Crasta et al., 2018). Beta-band oscillations help the brain perceive the rhythmicity of external stimuli. When humans need to synchronize with a specific rhythm, beta oscillations assist in adapting perception and actions to align with the rhythm (Fujioka et al., 2015). Studies have shown that while listening to a regular musical beat, beta activity energy decreases after each tone is heard (Comstock et al., 2021). Moreover, the rate of induced beta-band energy increase at the onset of the expected stimulus depends on the rhythm of the stimulus, whereas beta decrease after tone onset is consistent across multiple rhythms (Fujioka et al., 2009). This study primarily explored beta activity in motor areas. The results demonstrate that temporal factors and stimulus conditions significantly affect the beta-band energy in the motor area during SMS. However, the beta-band energy with different stimulation conditions all increased before the stimulus appeared and decreased afterward. This dynamic modulation process aligns with previous observations in SMS with visual and auditory stimuli containing only temporal information (Comstock et al., 2021; Fujioka et al., 2009). Our findings suggest that beta-band oscillations also play a modulatory role in SMS involving rhythmic stimuli containing spatial information. SMS across different stimulation conditions may all depend on the motor cortex, with beta-band modulation playing a crucial role in SMS.

The present study employed EEG-EMG coherence to examine the dynamic changes in CMC during SMS with rhythmic stimuli containing spatial information vs. those containing only temporal information. The results indicated that SMS across all stimulation conditions induced dynamic modulation of beta-band CMC in the contralateral corticomotor area. Specifically, CMC gradually decreased following the tapping action and gradually increased shortly before tapping. Previous research has highlighted the critical role of beta-band neural oscillations in cortical-muscular coupling, which underlies SMS, and has identified beta-band CMC as a potential mechanism for motor entrainment (Varlet et al., 2020; Nijhuis et al., 2021). Our study extends these findings by demonstrating that dynamic CMC modulation occurs during SMS regardless of whether the rhythmic stimulus contains spatial information. Additionally, we observed that stimulus modality affected the timing of the CMC peak. The CMC peak appeared before the stimulus in response to auditory stimuli, and after the stimulus for visual and auditory-visual stimuli, which aligns with the timing of the tapping. Behavioral data showed that tapping preceded the stimulus for auditory stimuli, while it lagged the stimulus for visual and auditory-visual stimuli during SMS. This observation is consistent with the study by Yoshida et al. (2017), suggesting that the dynamic modulation of CMC is primarily driven by motor processes. Furthermore, CMC appears to increase during finger extension and decrease during finger flexion, supporting the hypothesis that CMC plays a key role in stabilizing motion (Reyes et al., 2017). The study by Nijhuis et al. (2021) further demonstrated that CMC modulation in the β-band (14–38 Hz) of the contralateral cortical motor area is selectively time-locked to a tap performed synchronously with the stimulus. The dynamic modulation of beta-band oscillations between EEG and EMG likely contributes to stable tapping during SMS, and this modulation is present across different stimulation modalities and whether the stimuli contain spatial information. These findings underscore the critical role of beta-band neural oscillations in cortico-muscular coupling.

In addition, the present study assessed within-cortical connectivity in the beta-band using DTF to further investigate differences in cortical processing mechanisms during SMS with rhythmic stimuli that either include or exclude spatial information. Beta-band brain oscillations are closely associated with motor control (Crasta et al., 2018). By analyzing brain connectivity within this frequency band, we can gain insights into the brain mechanisms underlying SMS and identify the coordination between brain regions involved in SMS. Our findings indicate that compared to stimuli containing only temporal information, those containing spatial information exhibited higher levels of within-cortical connectivity between the motor cortex and other cortical areas during SMS. The motor cortex is known to play a role in the perception of auditory or visual spatial information (Arnott et al., 2004; Bizley and Cohen, 2013), which suggests that stimuli with spatial information may enhance interactions between the motor cortex and other cortical regions. Moreover, the information output flow from the motor cortex was significantly higher than the input flow, indicating that during SMS, the motor cortex primarily transmits information to other cortical areas. However, no significant differences were observed in the connectivity level of the contralateral motor cortex during SMS across different stimulus types. Future studies utilizing equipment with higher spatial resolution to record brain responses during SMS under various stimulation conditions may help to further elucidate the differences in interregional interactions during SMS with rhythmic stimuli that either include or exclude spatial information.

In conclusion, the present study examined the differences in SMS under six stimulation conditions: RAMS, RAS, RAVMS, RAVS, RVMS, and RVS. SMS performance was superior with auditory and auditory-visual stimuli compared to visual stimuli, and visual stimuli containing spatial information (i.e., RVMS) were also found to be superior to RVS. Additionally, the dynamic modulation of cortico-muscular coherence (CMC) in the beta band was consistent across all six stimulation conditions, highlighting the key role of beta-band (13 Hz−30 Hz) neural oscillations in cortical-muscular coupling and sensory-motor entrainment during SMS. Moreover, the study revealed that the information output from the motor cortex during SMS was greater than the information input, suggesting a proactive role of the motor cortex in coordinating sensory-motor integration. These findings enhance our understanding of the processing mechanisms involved in SMS with stimuli that contain spatial information. However, this study focused primarily on SSEP and dynamic responses during SMS with rhythmic sensory stimuli. Future research will delve deeper into the differences in brain perception among three conditions: rhythmic stimulation only, motor activity only, and SMS with rhythmic stimuli. This will further elucidate the brain processing mechanisms involved in SMS with stimuli that contain both spatial and temporal information.

## Data Availability

The raw data supporting the conclusions of this article will be made available by the authors, without undue reservation.
